# Association between GLP-1 RAs and DPP-4 inhibitors with biliary disorders: pharmacovigilance analysis

**DOI:** 10.3389/fphar.2025.1509561

**Published:** 2025-02-17

**Authors:** Long He, Jinwei Li, Xiong Cheng, Li Luo, Yilan Huang

**Affiliations:** ^1^ Department of Pharmacy, The Affiliated Hospital of Southwest Medical University, Luzhou, China; ^2^ School of Pharmacy, Southwest Medical University, Luzhou, China; ^3^ Department of Obstetrics and Gynecology, West China Second University Hospital, Sichuan University, Chengdu, China; ^4^ Key Laboratory of Birth Defects and Related Diseases of Women and Children, Sichuan University, Ministry of Education, Chengdu, China

**Keywords:** GLP-1 receptor agonists, DPP-4 inhibitors, FAERS, adverse events, biliary disorders, pharmacovigilance

## Abstract

**Background and Aims:**

Incretin-based therapies, including glucagon-like peptide 1 receptor agonists (GLP-1 RAs) and dipeptidyl peptidase-4 (DPP-4) inhibitors, are essential treatments in diabetes management due to their efficacy in glycemic control and the additional benefits of GLP-1 RAs, which include cardiovascular and renal protection. However, concerns about potential associations with biliary disorders necessitate ongoing pharmacovigilance. This study analyzes the link between these drugs and biliary adverse events (AEs) using the FDA Adverse Event Reporting System (FAERS) to enhance clinical safety.

**Methods:**

We extracted AE data for GLP-1 RAs and DPP-4 inhibitors from FAERS between Q1 2013 and Q1 2024 using OpenVigil 2.1. Analytical methods such as the Reporting Odds Ratio (ROR), Proportional Reporting Ratio (PRR), Bayesian Confidence Propagation Neural Network (BCPNN), and Empirical Bayesian Geometric Mean (EBGM) were employed to assess AE risk.

**Results:**

A search of biliary disorders by standard MedDRA analytical queries (SMQs) identified 2,215 reports of biliary AEs, with 1,709 related to GLP-1 RAs and 506 to DPP-4 inhibitors. DPP-4 inhibitors showed a significant association with biliary disorders (ROR, 3.09; 95% CI, 2.83–3.37), particularly sitagliptin (ROR, 3.46; 95% CI, 3.13–3.83). Although the overall association for GLP-1 RAs (ROR, 1.60; 95% CI, 1.52–1.68) was not significant, semaglutide (ROR, 4.06; 95% CI, 3.76–4.39) and liraglutide (ROR, 3.88; 95% CI, 3.50–4.29) indicated a notable risk. The SMQ subgroup analyses of sitagliptin, semaglutide, and liraglutide with the SMQ subgroup categories of “biliary tract disorders,” “gallbladder related disorders,” “gallstone related disorders,” and “infectious biliary disorders’ demonstrated a statistically significant correlation. Notably, liraglutide, alogliptin, sitagliptin, and linagliptin were linked to “biliary malignant tumors” with statistical significance. The proportion of serious outcomes was higher for DPP-4 inhibitors (n = 389, 76.88%) compared to GLP-1 RAs (n = 881, 51.55%).

**Conclusion:**

DPP-4 inhibitors are potentially linked to biliary disorders, warranting vigilance. While the overall association for GLP-1 RAs was not significant, specific drugs like semaglutide, liraglutide, and sitagliptin showed concerning signals, suggesting a need for heightened awareness among clinicians regarding the risk of biliary AEs.

## 1 Introduction

Diabetes, a significant global health challenge, is anticipated to reach $1.054 trillion in healthcare costs by 2045, with Type 2 Diabetes Mellitus (T2DM) affecting an estimated 783 million individuals (Sun et al., 2022). T2DM management necessitates a multifaceted strategy, including lifestyle modifications and pharmacological interventions (ElSayed et al., 2023). Among the medications, glucagon-like peptide 1 receptor agonists (GLP-1 RAs) and dipeptidyl peptidase-4 (DPP-4) inhibitors play pivotal roles. DPP-4 inhibitors manage blood sugar by extending the action of GLP-1, offering the benefits of oral administration, minimal side effects, and a low risk of hypoglycemia (Kasina et al., 2023). GLP-1 RAs enhance insulin secretion, suppress glucagon, slow gastric emptying, and reduce blood sugar, also decreasing appetite and weight (Collins and Costello, 2024). They provide cardiovascular and renal protection (Neuen et al., 2024), particularly benefiting obese T2DM patients or those with complications (Davies et al., 2022; ElSayed et al., 2023), making them valuable for individuals treated with GLP-1 RAs. However, the safety profile of these drugs, particularly their potential links to pancreatitis and biliary tract disease, remains a subject of concern within the medical community.

Numerous studies have investigated the relationship between DPP-4 inhibitors and the risk of biliary diseases. A meta-analysis and systematic review encompassing 82 randomized controlled trials revealed a significant association between DPP-4 inhibitors and an increased risk of cholecystitis, while no such association was found with an increased risk of cholecystolithiasis or other biliary diseases (He et al., 2022a). Another study, involving 75 randomized controlled trials with a total of 97,150 participants, demonstrated a statistically significant increase in the risk of cholecystitis among patients using DPP-4 inhibitors, yet it did not identify any significant associations between DPP-4 inhibitors and the risks of cholecystolithiasis, cholangitis, choledocholithiasis, or biliary colic (Yu et al., 2022). However, a cohort study involving 71,369 patients failed to find conclusive evidence that DPP-4 inhibitors significantly elevate the risk of bile duct and gallbladder diseases (Faillie et al., 2016). Regarding GLP-1RAs, earlier retrospective study suggested a potential link between the use of GLP-1RAs and an increased risk of gallbladder or biliary diseases (Faillie et al., 2016). Several randomized controlled trials (RCTs) also observed an increased incidence of cholecystitis or cholelithiasis in users compared to placebo controls (Roux et al., 2017; Lundgren et al., 2021; Wadden et al., 2021). A meta-analysis incorporating 76 randomized clinical trials indicated an association between the use of GLP-1RAs and an increased risk of gallbladder or biliary diseases, particularly when higher doses, longer durations, and weight loss were involved (He et al., 2022b). Nevertheless, it remains unclear whether the associated risks are similar across different GLP-1RA medications. Based on the available evidence, definitive conclusions cannot be drawn regarding the correlation between DPP-4 inhibitors and GLP-1RAs with the risk of biliary diseases.

Liyun He et al. have investigated the potential association between GLP-1 RAs, DPP-4 inhibitors, and biliary diseases utilizing the Food and Drug Administration Adverse Event Reporting System (FAERS) database. Their study primarily concentrated on overall usage trends of DPP-4 inhibitors and provided the usage proportions of specific drugs, such as sitagliptin. However, it did not conduct detailed analyses of each DPP-4 inhibitor or each GLP-1 RA. Furthermore, their research did not include an analysis of adverse biliary tumor events, nor did it provide detailed stratified data by gender and age (He et al., 2023). To further expand on existing research and provide a more granular safety assessment, our study conducted a comprehensive analysis of individual drug reports in the FAERS database using various statistical methods, including reporting odds ratio (ROR), proportional reporting ratio (PRR), Bayesian Confidence Propagation Neural Network (BCPNN), and Empirical Bayes Geometric Mean (EBGM). Our research not only covered trends in adverse biliary tumor events but also performed detailed stratified analyses by gender and age, offering insights for clinical treatment drug selection.

## 2 Materials and methods

### 2.1 Data sources

The FAERS database is a comprehensive repository of adverse event (AE) reports, medication error administration reports, and product quality complaints. It is a valuable resource for pharmacovigilance research, drawing data from a diverse range of sources, including health professionals, pharmaceutical manufacturers, attorneys, and individual patients. The FAERS database classifies AEs using standardized Medical Dictionary for Regulatory Activities (MedDRA) terms. This pharmacovigilance study utilized data extracted from the free pharmacovigilance tool OpenVigil 2.1, with adverse event reports imported from the FAERS database (Böhm et al., 2016).

### 2.2 Study procedure

**FIGURE 1:**
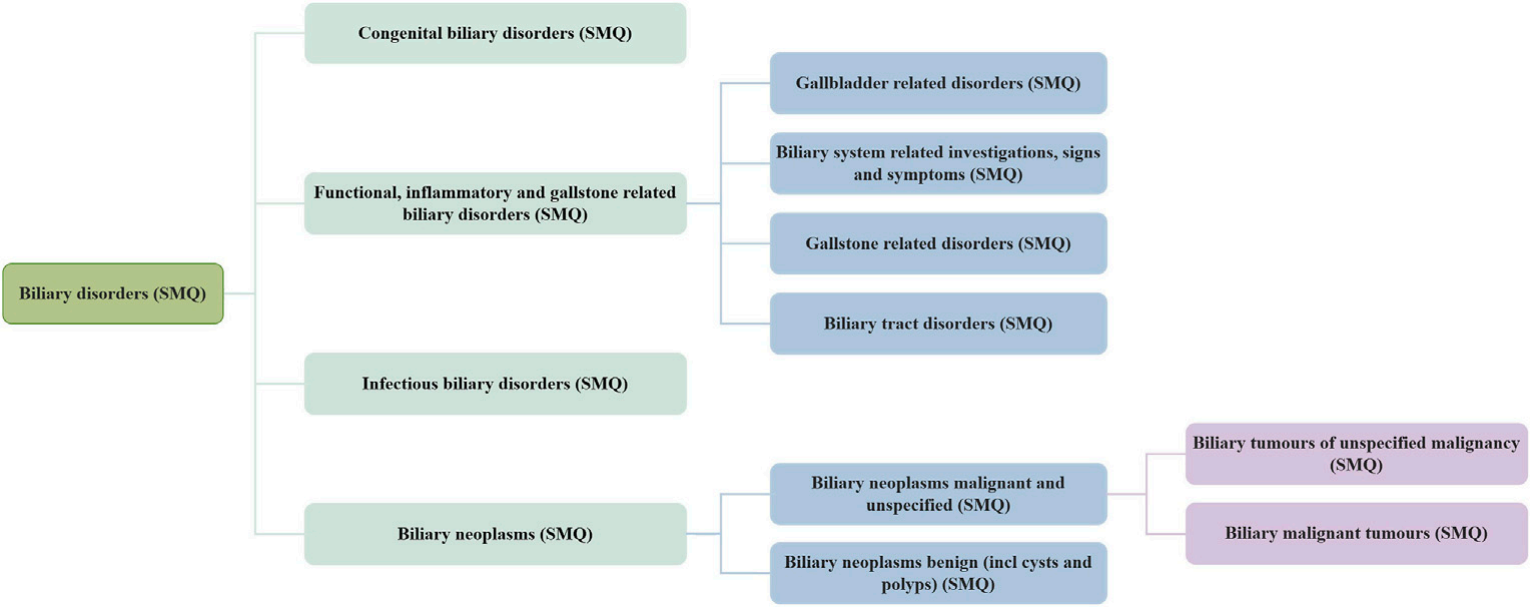
Grading relationships between SMQs associated with biliary disorders.

OpenVigil 2.1 (OpenVigil 2.1-MedDRA (stratoserver.net)), a pharmacovigilance tool embedded in MedDRA version 24.0, provides standard MedDRA analytical queries (SMQs) searching to facilitate the exploration of meaningful broader categories representing specific medical conditions or areas of interest. In this study, the hierarchical structure is displayed in Figure 1, and the list of SMQs retrieved is provided in the Supplementary Table S1. We extracted GLP-1 RAs (exenatide, liraglutide, dulaglutide, lixisenatide, semaglutide, tirzepatide) and DPP-4 inhibitors (linagliptin, alogliptin, saxagliptin, sitagliptin) from Q1 2013 to Q1 2024 for all AEs classified as biliary disorders (SMQ), with the drug role selecting the primary suspect drug (PS).

### 2.3 Data organisation

Some duplication of information exists in the extracted raw data. This can be excluded by two guidelines (Chen et al., 2024):

1. When the reported identifier codes (CASE_ID) are the same, retain the entry with the most recent FDA acceptance date (FDA_DT).
2. When CASE_ID and FDA_DT are the same, retain the entry with the higher identifier code.

The dates of reporting for all reports about both drug classes were collated and uploaded to an online mapping tool (https://www.chiplot.online/) to analyze trends in AE reporting. In addition, serious outcome statistics and analyses were performed for each drug. It should be noted that serious outcomes included death, Life-Threatening, Hospitalization-Initial or Prolonged, Disability, and Congenital Anomaly.

### 2.4 Statistical analysis

The AE reports were analyzed for signal detection using two-by-two contingency tables (Table 1) and four algorithms: the ROR, the PRR, the BCPNN, and the EBGM (shown in Supplementary Table S2). A positive AE signal was identified when it met the thresholds for all four methods (ROR: n ≥ 3, lower limit of 95% CI > 1; PRR: χ2 ≥ 4, lower limit of 95% CI > 1; EBGM: EBGM05 (EBGM05 denotes the lower bound of 95% CI) > 2; BCPNN: IC025 (IC025 denotes the lower bound of 95% CI) > 0). Data analysis was conducted using Navicate (version 16), Microsoft Excel (version 2021), and SPSS (version 27.0.1), with results visualized in Grighpade (version 9.5).

**TABLE 1:** Fourfold table of disproportionality analyses.

Drug	Target adverse events reported	Other adverse events reported	Summation
Target drugs	a	b	a+b
Other drugs	c	d	c+d
Summation	a+c	b+d	a+b+c+d

## 3 Descriptive analysis

A total of 149,349 AE reports for GLP-1 RAs and 23,022 for DPP-4 inhibitors were collected. After screening for biliary disorder AEs, 1,709 reports were identified for GLP-1 RAs and 506 for DPP-4 inhibitors (Table 2).

**TABLE 2:** Reported characteristics of biliary disorders associated with GLP-1RAs and DPP-4 inhibitors.

Characteristics		GLP-1 RA (N = 1,709)	DPP-4 inhibitors (N = 506)
Drug, n (%)			
	Exenatide	142 (8.31%)	
	Liraglutide	384 (22.47%)	
	Dulaglutide	290 (16.97%)	
	Lixisenatide	12 (0.70%)	
	Semaglutide	655 (38.33%)	
	Tirzepatide	226 (13.22%)	
	Alogliptin		13 (2.57%)
	Linagliptin		79 (15.61%)
	Sitagliptin		382 (75.49%)
	Saxagliptin		32 (6.32%)
Gender, n (%)			
	Male	647 (37.86%)	256 (50.59%)
	Female	944 (55.24%)	218 (43.08%)
	Not specified	4 (0.23%)	1 (0.20%)
	Unknown	114 (6.67%)	31 (6.13%)
Age, n (%)			
	<19	8 (0.47%)	0 (0)
	19–45	206 (12.05%)	24 (4.74%)
	46–65	466 (27.27%)	79 (15.61%)
	>65	371 (21.71%)	115 (22.73%)
	Unknown	658 (38.50%)	288 (56.92%)
	Median (years)	60 (49–69)	66 (55–77)
Outcome, n (%)			
	Death	67 (3.92%)	173 (34.19%)
	Disability	11 (0.64%)	2 (0.40%)
	Hospitalization	730 (42.72%)	190 (37.55%)
	Life-Threatening	66 (3.86%)	27 (5.34%)
	Other serious (Important medical event)	585 (34.23%)	86 (16.80%)
	Required intervention to prevent permanent impairment/damage	6 (0.35%)	0 (0.00%)
	Congenital anomaly	1 (0.06%)	0 (0.00%)
	Unknown	243 (14.22%)	29 (5.73%)
Country(ranking), n (%)			
	No.1	US: 1104 (64.60%)	US: 292 (57.71%)
	No.2	JP: 88 (5.15%)	JP: 64 (12.65%)
	No.3	GB: 66 (3.86%)	FR: 20 (3.95%)

N, the number of AE reports related to biliary disorders.

The GLP-1 RA with the highest number of reports was semaglutide, accounting for 655 cases (38.33%). Biliary disorders were more commonly reported in females (55.24%) than males (37.86%), with the majority of patients aged 46–65 years (27.27%). The median age was 60 years, and the most frequently reported serious outcome was hospitalization (42.72%), predominantly reported by individuals in the US (64.60%). For DPP-4 inhibitors, sitagliptin had the most reports (75.49%), with males slightly more affected (50.59%). The age distribution was more pronounced among patients aged 65 years (22.73%) and above. The median age was 66 years, with hospitalization (37.55%) and death (34.19%) being the most frequently reported serious outcomes, the latter largely attributed to sitagliptin (97.69%). Similar to GLP-1 RAs, the majority of DPP-4 inhibitor AE reports originated from the US (57.71%).

**FIGURE 2:**
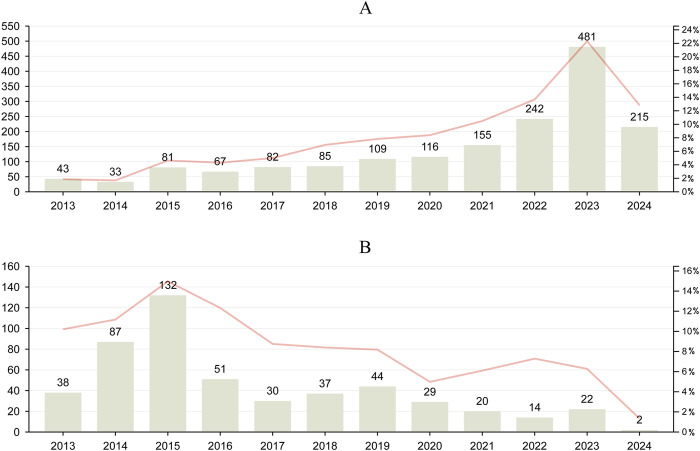
Trends in reported biliary disorders and overall AEs associated with GLP-1 RAs and DPP-4 inhibitors from Q1 2013 to Q1 2024. **(A)**, the report trend of GLP-1RAs. **(B)**, the report trend of DPP-4 inhibitors. Line graphs were used to characterise trends in all AE reports for GLP-1 RAs and DPP-4 inhibitors.

The number of reports was analyzed to identify trends, which were then compared with the reporting of AE reports for biliary disorders (Figure 2). The number of adverse events associated with GLP-1 RAs has been on the rise since 2013, with biliary disorder AE reports reaching 481 in 2023. This trend is expected to continue throughout 2024, with an estimated increase in the number of AEs. For DPP-4 inhibitors, the overall number of AE reports and biliary disorder AE reports demonstrated an upward and then a downward trend, reaching a peak in 2015 with 132 biliary disorder AE reports. Despite minor fluctuations, the number of AE reports for DPP-4 inhibitors has remained low in recent years.

**FIGURE 3:**
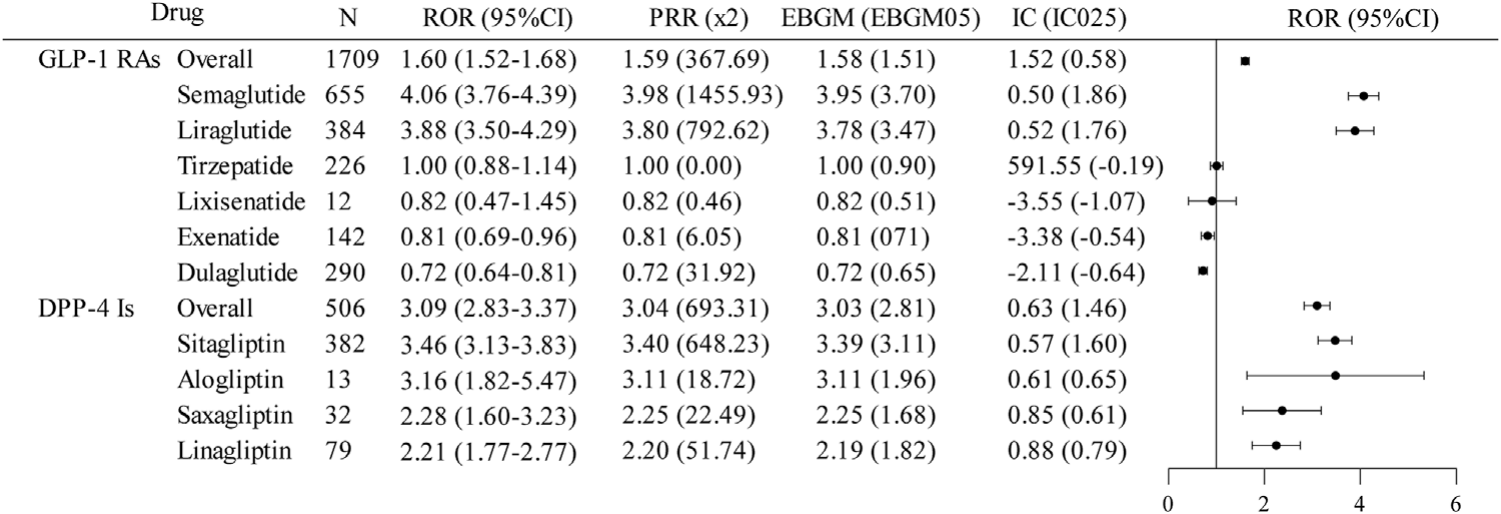
Disproportionate analysis of AE reports of biliary disorders associated with GLP-1 RAs and DPP-4 inhibitors. GLP-1RAs, glucagon-like peptide-1 receptor agonists; DPP-4 Is, dipeptidyl peptidase-4 inhibitors; N, the report number; ROR, the reporting odds ratio; PRR, the proportional reporting ratio; IC, the information component; EBGM, the empirical Bayes geometric mean; CI, confidence interval; 95% CI, two-sided for ROR; x2, chi-squared; IC025 and EBGM05 lower one-sided for IC, and EBGM.

The results for the SMQ of biliary disorders (Figure 3) showed that only DPP-4 inhibitors indicated a positive signal for biliary disorder AEs overall. The overall signal intensity of GLP-1 RAs [ROR (95%CI) = 1.60 (1.52–1.68); PRR (χ2) = 1.59 (367.69); EBGM (EBGM05) = 1.58 (1.51); IC (IC025) = 1.52 (0.58)] was primarily attributable by semaglutide [ROR (95%CI) = 4.06 (3.76–4.39); PRR (χ2) = 3.98 (1455.93); EBGM (EBGM05) = 3.95 (3.70); IC (IC025) = 0.50 (1.86)] and, to a lesser extent, liraglutide [ROR (95%CI) = 3.88 (3.50–4.29); PRR (χ2) = 3.80 (792.62); EBGM (EBGM05) = 3.78 (3.47); IC (IC025) = 0.52 (1.76)], and the AE of biliary disorders was considered a positive signal for both drugs. In comparison, the other GLP-1 RAs exhibited a lower signal intensity [ROR (95%CI) ≤ 1). For DPP-4 inhibitors, all four drugs showed a strong association with biliary disorders (ROR (95%CI) > 1), but only sitagliptin (ROR (95%CI) = 3.46 (3.13–3.83); PRR (χ2) = 3.40 (648.23); EBGM (EBGM05) = 3.39 (3.11); IC (IC025) = 0.57 (1.60)] was considered a positive signal.

**FIGURE 4:**
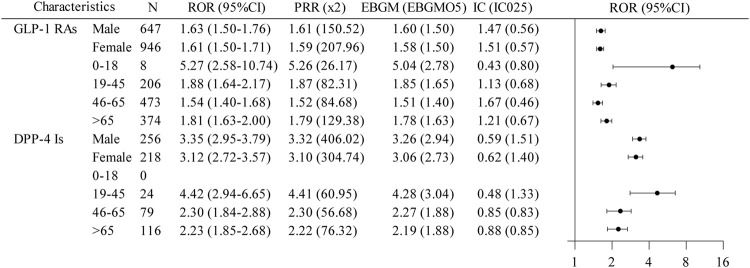
A disproportionate subgroup analysis of AE reports of biliary disorders associated with GLP-1 RAs and DPP-4 inhibitors. GLP-1RAs, glucagon-like peptide-1 receptor agonists; DPP-4 Is, dipeptidyl peptidase-4 inhibitors; N, the report number; ROR, the reporting odds ratio; PRR, the proportional reporting ratio; IC, the information component; EBGM, the empirical Bayes geometric mean; CI, confidence interval; 95% CI, two-sided for ROR; x2, chi-squared; IC025 and EBGM05, lower one-sided for IC and EBGM.

To further elucidate the association between GLP-1 RAs and DPP-4 inhibitors with biliary disorders, we analyzed data across various gender and age groups (Figure 4). For GLP-1 RAs, patients aged 0–18 years showed a notably higher signal intensity for biliary disorder AEs, meeting the thresholds for all four analytical methods. Conversely, DPP-4 inhibitors lacked data for the 0–18 years age group, but both males and females in the 19–45 years age bracket for DPP-4 inhibitors displayed a positive AE signal for biliary disorder AEs. The 95%CI of RORs exceeded 1 for all assessable subgroups of GLP-1 RAs and DPP-4 inhibitors, suggesting a potential statistical link to the risk of biliary disorders, aligning with the general signal strength for these conditions.

The analysis of the SMQs segmentation structure revealed drugs with a positive signal or the highest EBGM values, as detailed in Table 3. For GLP-1 RAs, semaglutide was notably associated with a significant risk of “biliary tract disorders” (n = 188) and “infectious biliary disorders” (n = 196). Liraglutide was identified as posing the highest potential risk for “biliary malignant tumors” (n = 19) and was also linked to common GLP-1 RA adverse events, including “gallbladder related disorders” (n = 300) and “gallstone related disorders” (n = 176), both of which were statistically significant. Regarding DPP-4 inhibitors, sitagliptin was associated with the highest number of adverse events across the top five SMQs, indicating the greatest potential risk. These included “gallbladder related disorders” (n = 212), “biliary tract disorders” (n = 188), “gallstone related disorders” (n = 120), “biliary system-related investigations, signs and symptoms” (n = 94), and “infectious biliary disorders” (n = 49). Alogliptin, sitagliptin, and linagliptin were identified as posing the highest potential risk for “biliary malignant tumors,” with ROR (95%CI) of 17.97 (95% CI: 5.77–55.90), 6.24 (4.30–9.06), and 6.25 (3.25–12.03), respectively; EBGM (EBGM05) values were 17.86 (6.91), 6.18 (4.53), and 6.22 (3.60), all of which were statistically significant.

**TABLE 3:** Results of disproportionate analysis of AEs associated with biliary disorders of GLP-1 RAs and DPP-4 inhibitors at the SMQ level.

Characteristics			N	ROR (95%CI)	EBGM (EBGM05)	Positive signal
	SMQ	Drug				
GLP-1 RAs	Biliary malignant tumours	Overall	62	1.43 (1.11–1.84)	1.42 (1.15)	No
		Liraglutide	19	4.69 (2.99–7.36)	4.66 (3.19)	Yes
	Biliary neoplasms benign (incl cysts and polyps)	Overall	4	0.89 (0.33–2.39)	0.89 (0.39)	No
		Liraglutide	2	4.79 (1.19–19.25)	4.77 (1.49)	No
	Biliary system related investigations, signs and symptoms	Overall	255	0.56 (0.49–0.63)	0.56 (0.51)	No
		Semaglutide	75	1.08 (0.86–1.35)	1.08 (0.89)	No
	Biliary tract disorders	Overall	448	1.03 (0.94–1.13)	1.03 (0.96)	No
		Semaglutide	188	2.86 (2.48–3.30)	2.83 (2.51)	Yes
		Liraglutide	97	2.4 (1.97–2.93)	2.39 (2.02)	Yes
	Biliary tumours of unspecified malignancy	Overall	1			No
		Liraglutide	1			No
	Congenital biliary disorders	Overall	3	0.17 (0.06–0.53)	0.17 (0.07)	No
		Liraglutide	2			No
	Gallbladder related disorders	Overall	1,222	3.48 (3.28–3.68)	3.33 (3.18)	Yes
		Liraglutide	300	9.01 (8.03–10.11)	8.74 (7.94)	Yes
		Semaglutide	479	8.86 (8.09–9.71)	8.54 (7.91)	Yes
		Tirzepatide	180	2.38 (2.05–2.75)	2.36 (2.09)	Yes
	Gallstone related disorders	Overall	658	4.65 (4.30–5.04)	4.40 (4.11)	Yes
		Liraglutide	176	12.92 (11.12–15.01)	12.56 (11.08)	Yes
		Semaglutide	272	12.32 (10.91–13.91)	11.88 (10.73)	Yes
	Infectious biliary disorders	Overall	425	2.67 (2.42–2.94)	2.60 (2.40)	Yes
		Semaglutide	196	8.03 (6.97–9.25)	7.84 (6.96)	Yes
		Liraglutide	85	5.62 (4.54–6.96)	5.56 (4.64)	Yes
DPP-4 Is	Biliary malignant tumours	Overall	42	6.34 (4.68–8.61)	6.26 (4.85)	Yes
		Alogliptin	3	17.97 (5.77–55.90)	17.86 (6.91)	Yes
		Sitagliptin	28	6.24 (4.30–9.06)	6.18 (4.53)	Yes
		Linagliptin	9	6.25 (3.25–12.03)	6.22 (3.60)	Yes
	Biliary neoplasms benign (incl cysts and polyps)	Overall	5	7.35 (3.03–17.78)	7.24 (3.45)	Yes
		Sitagliptin	3	6.49 (2.08–20.25)	6.44 (2.48)	No
	Biliary system related investigations, signs and symptoms	Overall	134	1.92 (1.62–2.28)	1.91 (1.66)	No
		Sitagliptin	94	2.00 (1.63–2.45)	1.99 (1.68)	No
	Biliary tract disorders		239	3.62 (3.19–4.12)	3.57 (3.21)	Yes
		Sitagliptin	188	4.23 (3.66–4.88)	4.17 (3.69)	Yes
	Biliary tumours of unspecified malignancy	Overall	6	27.88 (12.21–63.68)	26.23 (13.14)	Yes
		Sitagliptin	6	41.36 (18.11–94.48)	38.88 (19.48)	Yes
	Congenital biliary disorders	Overall	0			No
	gallbladder related disorders	Overall	269	4.85 (4.30–5.47)	4.76 (4.30)	Yes
		Sitagliptin	212	5.66 (4.94–6.49)	5.56 (4.96)	Yes
		Alogliptin	7	5.00 (2.37–10.54)	4.95 (2.65)	Yes
		Saxagliptin	16	3.36 (2.05–5.49)	3.33 (2.21)	Yes
		Linagliptin	34	2.81 (2.00–3.94)	2.79 (2.11)	Yes
	Gallstone related disorders	Overall	156	6.89 (5.88–8.08)	6.76 (5.92)	Yes
		Sitagliptin	120	7.84 (6.55–9.40)	7.71 (6.63)	Yes
		Alogliptin	5	8.73 (3.62–21.06)	8.66 (4.14)	Yes
		Saxagliptin	10	5.13 (2.75–9.55)	5.10 (3.03)	Yes
		Linagliptin	21	4.25 (2.76–6.52)	4.23 (2.95)	Yes
	Infectious biliary disorders	Overall	75	3.00 (2.39–3.76)	2.98 (2.46)	Yes
		Sitagliptin	49	2.90 (2.19–3.84)	2.88 (2.28)	Yes

GLP-1RAs, glucagon-like peptide-1, receptor agonists; DPP-4, is, dipeptidyl peptidase-4 inhibitors; N, the report number; SMQs, standard MedDRA, analytical queries; ROR, the reporting odds ratio; EBGM, the empirical Bayes geometric mean; CI, confidence interval; 95% CI, two-sided for ROR; EBGM05, lower one-sided for EBGM.

**FIGURE 5:**
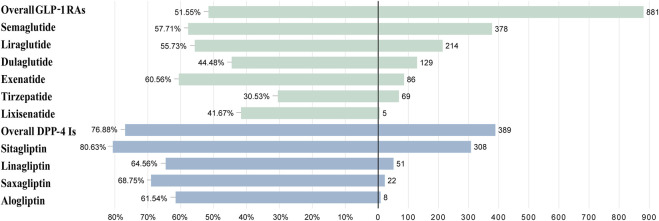
The case severe rate for GLP-1 RAs and DPP-4 inhibitors associated with biliary disorders.

The prognosis of AE reports was assessed using the serious outcome ratio. Please refer to Table 2; Figure 5 for details. There was a significant difference in the proportion of serious outcomes among different GLP-1 RAs (*p* < 0.01, Pearson’s χ2 = 49.77). Exenatide (n = 86, 60.56%) had the highest proportion of serious outcomes, while tirzepatide (n = 69, 30.53%) had the lowest. There was a notable discrepancy in the incidence of serious outcomes across different DPP-4 inhibitors (*p* = 0.011, Pearson’s χ2 = 11.16), with sitagliptin (n = 308, 80.63%) exhibiting the highest prevalence and alogliptin (n = 8, 61.54%) the lowest. DPP-4 inhibitors demonstrated a higher proportion of serious outcomes compared to GLP-1 RAs (*p* < 0.01, Pearson’s χ2 = 116.46).

## 4 Discussion

In this study, we employed the drug safety network tool OpenVigil 2.1 and applied disproportionate analysis to explore the statistical associations between GLP-1 RAs, DPP-4 inhibitors, and biliary diseases at the standardized MedDRA query (SMQ) level. This analysis was based on the extensive data from the FAERS database. The results showed a significant statistical correlation between DPP-4 inhibitors and AEs related to biliary diseases, while GLP-1 RAs exhibited strong signal intensity, suggesting a possible association with biliary diseases. In the disproportionate analysis of individual drugs, we further found significant correlations between liraglutide, semaglutide, and sitagliptin with biliary diseases, with these drugs being the main contributors to the overall signal intensity in their respective categories. Notably, compared to GLP-1 RAs, the use of DPP-4 inhibitors showed a higher correlation with more severe outcomes, including death, highlighting their potential risks.

Our findings showed the significant statistical association between sitagliptin [ROR 3.46; 95% CI 3.13–3.83; EBGM05 = 3.39 (3.11)]and biliary AEs, as well as the high proportion of severe outcomes (80.63%), which underscores its potential safety concerns. The study by LI et al. also indicated that, compared to other antidiabetic drugs, DPP-4 inhibitors led to an increased number of reports of gallbladder or biliary diseases, with sitagliptin accounting for up to 80% of the cases (He et al., 2023). Additionally, a cohort study by Shapiro et al. revealed that DPP-4 inhibitor users had a 46% increased risk of biliary diseases compared to SGLT-2 inhibitor users, with 4.3 events per 1000 person-years in the DPP-4 inhibitor group and 3.0 events in the control group, showing a statistically significant difference (HR 1.46, 95% CI 1.17–1.83) (Shapiro et al., 2024). Two meta-analyses in 2022 also confirmed that DPP-4 inhibitors significantly increase the risk of gallbladder/biliary diseases and cholecystitis (He et al., 2022a; Yu et al., 2022). The proportion of death outcomes of DPP-4 inhibitors was mutually confirmed with previous study (34.19% vs. 33.27%) (He et al., 2023), while GLP-1RAs was in a much lower proportion. There is no more evidence to explain such statistical results, suggesting that this is a potential research direction and needs to be verified by subsequent studies.

For GLP-1 RAs, our results indicated that although it did not show a direct correlation with biliary diseases overall, specific drugs such as semaglutide [ROR 4.06; 95%CI 3.76–4.39; EBGM05 = 3.95 (3.70)] and liraglutide [ROR 3.88; 95%CI 3.50–4.29; EBGM05) = 3.78 (3.47)] were significantly associated with an increased risk of biliary diseases. This is consistent with previous experimental findings (Lundgren et al., 2021; Wadden et al., 2021; Wilding et al., 2021). The systematic review by Liyun He et al. also found that the use of GLP-1 RAs was associated with an increased risk of cholelithiasis (RR 1.27; 95% CI 1.10–1.47), cholecystitis (RR 1.36; 95% CI 1.14–1.62), and biliary diseases (RR 1.55; 95% CI 1.08–2.22) (He et al., 2022b). Furthermore, studies by He et al. (2022b) and Yang et al. (2024) showed that higher doses, long-term treatment, and accompanying weight loss with GLP-1 RAs were associated with an increased risk of biliary diseases. FDA-approved semaglutide and liraglutide for weight loss in obese patients often require long-term and higher doses to achieve significant weight reduction (Rothberg et al., 2024), which may explain the association between semaglutide and liraglutide and the increased risk of biliary diseases. Therefore, when assessing the safety of GLP-1 RAs, factors such as drug specificity, dose, treatment duration, and use for weight loss should be considered to comprehensively understand their potential impact on biliary diseases.

The direct link between DPP-4 inhibitors and GLP-1 RAs with biliary tumors remains unclear in current research. Some studies have shown that these drugs do not significantly increase the risk of cholangiocarcinoma. However, our study has revealed potential associations between DPP-4 inhibitors, particularly alogliptin, linagliptin, and sitagliptin, with biliary tumors. In adverse event reports, alogliptin was associated with 3 cases of biliary malignancies, linagliptin with 9 cases, and their 95%CI for ROR and EBGM05 exceeded the signal detection thresholds, indicating a possible statistically significant correlation. The situation with sitagliptin is even more pronounced, with a total of 28 reported cases of biliary malignancies associated with its use. These data provide further support for the hypothesis that DPP-4 inhibitors may increase the risk of biliary tumors. The study by Abrahami et al. also suggested an increased risk of cholangiocarcinoma associated with the use of DPP-4 inhibitors and GLP-1 RAs, but the study involving GLP-1 RAs had a wide confidence interval, leading to some uncertainty in the conclusion (Abrahami et al., 2018). Other study examining the association between GLP-1 RAs and cholangiocarcinoma did not find a significant increase in risk with their use, although 26 cases were reported, failing to establish a statistically significant association between GLP-1 RA use and increased risk (Ueda et al., 2021). In our study, the EBGM05 values for GLP-1 RAs did not reach the preset thresholds, thus insufficient to establish a clear causal relationship between them and biliary tumors. However, the relatively high number of reports related to liraglutide, along with its elevated values [(ROR 4.69; 95%CI 2.99–7.36); EBGM05 = 4.66 (3.19)], may suggest a certain association with the occurrence of biliary malignancies, warranting further investigation.

Regarding the possible mechanisms of biliary diseases caused by these two types of drugs, existing studies have proposed multiple hypotheses. DPP-4 inhibitors may affect biliary health through various mechanisms, including enhancing the effects of incretins, influencing bile secretion, modulating the neural axis, and regulating inflammatory responses (Kasina et al., 2023). However, the specific mechanism between DPP-4 inhibitors and biliary diseases (especially biliary tumors) is currently unclear and requires further research to elucidate. On the other hand, GLP-1 RAs may alter the physiological state of the biliary tract by affecting gallbladder contraction and emptying function (Rehfeld et al., 2018; Collins and Costello, 2024). Studies have shown that the use of GLP-1 RAs is associated with gallbladder dysfunction and bile stasis (Faillie et al., 2016; He et al., 2022b). Additionally, GLP-1 RAs can regulate lipid metabolism, including cholesterol synthesis and excretion (Bu et al., 2024). They may increase cholesterol concentrations in bile by inhibiting lipid synthesis in the liver, thereby promoting stone formation (Monami et al., 2017; Nreu et al., 2020). Long-term use of GLP-1 RAs may lead to chronic inflammatory reactions in the biliary tract, potentially increasing the risk of malignancies.

It is crucial to acknowledge the limitations of this study. Firstly, the FAERS database, being an open-access spontaneous reporting system, is prone to biases in completeness and accuracy due to the reporting process and expertise of reporters. Secondly, our analysis on the OpenVigil platform focused solely on the correlation between the drug and adverse event (AE), disregarding drug-drug interactions when multiple medications are prescribed. Additionally, potential confounding factors such as diabetes and obesity, which increase the risk of biliary dysfunction, were not controlled for. While data mining techniques offer advantages in analyzing vast real-world datasets, it is imperative to recognize their inherent limitations. Our signal detection merely indicates a statistical correlation, necessitating further investigation for definitive causality. Nonetheless, our findings present novel perspectives and avenues for future research.

## 5 Conclusion

Our study highlights a substantial association between DPP-4 inhibitors, notably sitagliptin, and biliary disorders. Although GLP-1 RAs as a class do not exhibit a disproportionate correlation with biliary diseases, specific agents like semaglutide and liraglutide demonstrate marked signal intensity, suggesting a potential risk. Given these findings, clinicians must carefully consider patient-specific conditions and potential risks when prescribing these medications to ensure optimal patient safety and rational drug use. Future research should delve deeper into the specific mechanisms linking these drugs to biliary diseases and examine the impact of dosage and treatment duration on risk, thereby informing more personalized and safer medication guidance in clinical practice.

## Data Availability

The original contributions presented in the study are included in the article/Supplementary Material, further inquiries can be directed to the corresponding authors.

## Abbreviations

T2DM, Type 2 Diabetes Mellitus; GLP-1, RAs Glucagon-like peptide 1 receptor agonists; DPP-4, Dipeptidyl peptidase-4; RCTs, Randomized controlled trials; FAERS, Food and Drug Administration Adverse Event Reporting System; ROR, Reporting odds ratio; PRR, Proportional reporting ratio; BCPNN, Bayesian Confidence Propagation Neural Network; EBGM, Empirical Bayes Geometric Mean; AE, Adverse event; MedDRA, Medical Dictionary for Regulatory Activities; SMQs, Standard MedDRA analytical queries; PS, Primary suspect drug.
