# Supplementary material for: Association between GLP-1 RAs and DPP-4 inhibitors with biliary disorders: pharmacovigilance analysis
Source: Front Pharmacol. 2025 Feb 17;16:1509561. doi: 10.3389/fphar.2025.1509561 (PMC11878242; doi:10.3389/fphar.2025.1509561)
Supplement: Supplementary file 1 [file Table1.doc]

**Association between GLP-1 RAs and DPP-4 Inhibitors with biliary disorders: pharmacovigilance analysis**

Long He, ^1 , 2, †^ Jinwei Li, ^1, 2, †^ Xiong Cheng, ^1, 2^ Li Luo, ^3, 4, #, *^and Yilan Huang ^1, 2, #, *^

^1^ School of Pharmacy, Southwest Medical University, Luzhou, China

^2^ Department of Pharmacy, The Affiliated Hospital of Southwest Medical University, Luzhou, China

^3^ Department of Obstetrics and Gynecology, West China Second University Hospital, Sichuan University, Chengdu, China

^4^ Key Laboratory of Birth Defects and Related Diseases of Women and Children, Sichuan University, Ministry of Education, Chengdu, China

^†^ These authors share first authorship

^#^ Contributed equally.

^*^ Correspondence: Li Luo, [feigagasmile@163.com;](mailto:feigagasmile@163.com;) Yilan Huang, [hyl3160131@126.com](mailto:hyl3160131@126.com)

Supplymentary table 1

List of SMQs related to biliary diseases involved in the study.

| No. | SMQ |
| --- | --- |
| 1 | biliary malignant tumours (SMQ) |
| 2 | biliary neoplasms benign (incl cysts and polyps) (SMQ) |
| 3 | biliary system related investigations, signs and symptoms (SMQ) |
| 4 | biliary tract disorders (SMQ) |
| 5 | biliary tumours of unspecified malignancy (SMQ) |
| 6 | congenital biliary disorders (SMQ) |
| 7 | gallbladder related disorders (SMQ) |
| 8 | gallstone related disorders (SMQ) |
| 9 | infectious biliary disorders (SMQ) |

Supplymentary table 2

Four methods of disproportionality analysis.

| **Algorithms** | **Equation** | **Criteria** |
| --- | --- | --- |
| **ROR** | 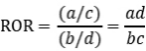  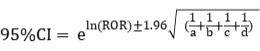 | a≥3, lower limit of 95%Cl＞1 |
| **PRR** | 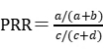  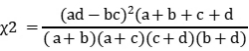 | a≥3, PRR≥2 |
| **BCPNN** | IC=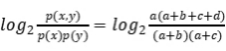  E(IC)=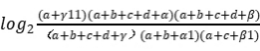  V(IC)=  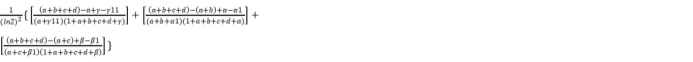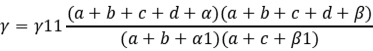  IC-2SD=E(IC)-2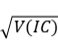  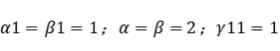 | 1.According to the strength of the signal  ①No signal(-):IC-2SD≤0;  ②Weak signal(+): 0＜IC-2SD≤1.5;  ③Medium signal(++): 1.5＜IC-2SD≤3;  ④Strong signal(+++): IC-2SD＞3  2.According to whether there is signal or not  a≥3, IC-2SD＞0 |
| **MGPS** | 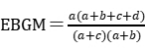  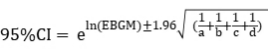 | EBGM_05_＞2 |
